# Cell therapy in patients with COVID-19 using Wharton’s jelly mesenchymal stem cells: a phase 1 clinical trial

**DOI:** 10.1186/s13287-021-02483-7

**Published:** 2021-07-16

**Authors:** Mahshid Saleh, Amir Abbas Vaezi, Rasoul Aliannejad, Amir Ali Sohrabpour, Seyedeh Zahra Fotook Kiaei, Mahdi Shadnoush, Vahid Siavashi, Leila Aghaghazvini, Batoul Khoundabi, Shahriyar Abdoli, Bahram Chahardouli, Iman Seyhoun, Neda Alijani, Javad Verdi

**Affiliations:** 1 Department of Applied Cell Sciences, School of Advanced Technologies in Medicine, Tehran University of Medical Sciences, Tehran, Iran; 2 Department of Internal Medicine, Alborz University of Medical Sciences, Karaj, Iran; 3 Department of Pulmonary and Critical Care, Shariati Hospital, Tehran University of Medical Sciences, Tehran, Iran; 4 Advanced Thoracic Research Center, Tehran University of Medical Sciences, Tehran, Iran; 5 Associate Professor of Gastroenterology and Hepatology, Liver and Pancreatobiliary Diseases Research Center, Digestive Disease Research Institute, Tehran University of Medical Sciences, Tehran, Iran; 6 Advanced Thoracic Research Centre, Tehran University of Medical Science, Tehran, Iran; 7 Department of Clinical Nutrition, Faculty of Nutrition & Food Technology, Shahid Beheshti University of Medical Sciences, Tehran, Iran; 8 Department of Clinical Pathology, Faculty of Veterinary Medicine, University of Tehran, Tehran, Iran; 9 Associate Professor, Department of Radiology, Shariati Hospital, Tehran University of Medical Sciences, Tehran, Iran; 10 Iran Helal Institute of Applied-Science and Technology, Research Center for Health Management in Mass Gathering, Red Crescent Society of the Islamic Republic of Iran, Tehran, Iran; 11 Pasteur Institute of Iran, National Cell Bank of Iran, Tehran, Iran; 12 Hematology, Oncology, and Stem Cell Transplantation Research Center, Tehran University of Medical Sciences, Tehran, Iran; 13 Department of Infectious Diseases, Shariati Hospital, Tehran University of Medical Sciences, Tehran, Iran

**Keywords:** COVID-19, WJ-MSC, Cell therapy

## Abstract

**Background:**

Mesenchymal stem cells (MSCs) have received particular attention because of their ability to modulate the immune system and inhibit inflammation caused by cytokine storms due to SARS-CoV-2. New alternative therapies may reduce mortality rates in patients with COVID19. This study aimed to assess the safety and efficacy of injecting intravenous Wharton’s jelly-derived MSCs in patients with COVID-19 as a treatment.

**Methods:**

In this study, five patients with severe COVID-19 were treated with Wharton’s jelly-derived mesenchymal stem cells (150 × 106 cells per injection). These patients were subject to three intravenous injections 3 days apart, and monitoring was done on days 0, 3, 6, and 14 in routine tests, inflammatory cytokines, and flow cytometry of CD4 and CD8 markers. A lung CT scan was performed on base and days 14 and 28. In addition, IgM and IgG antibodies against SARS-CoV-2 were measured before and after treatment.

**Results:**

The results showed that IL-10 and SDF-1 increased after cell therapy, but VEGF, TGF-β, IFN-γ, IL-6, and TNFα decreased. Routine hematology tests, myocardial enzyme tests, biochemical tests, and inflammation tests were performed for all patients before and after cell therapy on base and days 3, 6, and 14, which indicated the improvement of test results over time. COVID-19 antibody tests rose in 14 days after WJ-MSC injection. The total score of zonal involvement in both lungs was improved.

**Conclusions:**

In patients, the trend of tests was generally improving, and we experienced a reduction in inflammation. No serious complications were observed in patients except the headache in one of them, which was resolved without medication. In this study, we found that patients with severe COVID-19 in the inflammatory phase respond better to cell therapy. More extensive clinical trials should be performed in this regard.

**Trial registration:**

IRCT, IRCT20190717044241N2. Registered April 22, 2020.

**Supplementary Information:**

The online version contains supplementary material available at 10.1186/s13287-021-02483-7.

## Introduction

Coronavirus disease 2019 (COVID-19) has spread worldwide and was first detected in Wuhan, China, in December 2019 [1]. The virus has been widely distributed in different geographical areas [2]. SARS-CoV-2 primarily involves the respiratory system in addition to affecting other organs. Symptoms associated with lower respiratory tract infections, including fever, dry cough, and shortness of breath, have been reported in early case series isolated from Wuhan, China [3] Signs such as headache, dizziness, general weakness, vomiting, and diarrhea were also observed [4]. According to 2020 guidelines of the WHO, severe coronavirus disease is defined as follows: an adolescent or adult with clinical signs of pneumonia (fever, cough, shortness of breath, rapid breathing) plus one of the following signs: respiratory rate > 30 breaths per minute; severe respiratory distress; or SpO2 < 90% in ambient air [5].

At present, there is no specific drug to treat COVID19. The pathogenesis of HcoV19 is mediated through the detection of the ACE2 receptor by the spike protein of this virus [6–8]. Unfortunately, the ACE2 receptor is abundantly present in human cells, especially alveolar type II (ATII) and capillary endothelium. Immune cells such as B and T lymphocytes as well as macrophages in the bone marrow, lymph nodes, thymus, and spleen are negative for ACE2 [9].

Up to now, adjuvant therapeutic strategies such as corticosteroid-mediated reduction of inflammation, treatment of congestion using plasma, administration of antibiotics to treat secondary bacterial infections, nonspecific antiviruses, and so forth have not been effective in severe cases of COVID-19. The main reason for these treatments’ failure is the cytokine storm created in the lungs by the virus [10, 11]. SARS-CoV-2 can provoke severe cytokine storms in the lungs, including IL-2, IL-6, IL-7, GCSF, IP10, MCP1, MIP1A, and TNFα, followed by edema, defective respiration, acute respiratory distress syndrome, acute heart damage, secondary infection [12], and eventually death [13]. More than 44 clinical trials on cell therapy in patients with COVID-19 have been registered at www.clinicaltrials.gov, www.chictr.org, and www.irct.ir and are being conducted. At present, different cells are used worldwide, including NK cells, T cells, and MSC cells of allogeneic and autologous origin from various tissues such as adipose, placenta, umbilical cord, Wharton’s jelly, dental pulp, and menstrual blood [14]. The safety and efficacy of mesenchymal stem cells in reducing inflammatory lung disease have been indicated in animal models [15]. In human clinical trials, all reports indicated that stem cell injections were safe. Although the effects of cell therapy are not uniform, positive effects of cell therapy have been expressed in some cases but not in others [16]. In general, phase I and II clinical trials provide early immune results in patients with bronchopulmonary dysplasia (BPD), asthma, chronic obstructive pulmonary disease (COPD) [17], idiopathic pulmonary fibrosis (IPF) [18–21], and in patients with acute lung injury (ALI) [22].

Mesenchymal stem cells have strong immunomodulatory capabilities and may help treat and attenuate cytokines. MSCs have been implicated in several clinical trials in GVHD and SLE [23]. Many clinical trials in different conditions associated with these cells are currently performed for the disease [24]. MSCs have two main effects, namely immunomodulation [25] and differentiation [26]. Meanwhile, Wharton’s jelly-derived MSCs are easily and non-invasively isolated from neonatal tissues [27] and show a higher pluripotential property compared to other sources of MSCs [28, 29]. Various studies have revealed that WJ-MSCs affect almost all immune cells and suppress CD3, CD8, and CD4 T cells [30]. These cells play a vital role in modulating the immune system by secreting large amounts of cytokines like IL-10, TGF-b, IL-6, and VEGF [31]. In a study by Zhang et al., 1 × 106 of WJ-MSC cells were injected in one dose for a critically severe-type patient. The patient underwent various treatments, such as antiviral therapies, plasma exchange, corticosteroids, and so on. Unfortunately, the patient’s condition exacerbated after a few days, during which the treatment team used WJ-MSC cells for the patient. This study found that WJ-MSC cells could be an ideal and practical option for treating COVID19 patients [32]. The immunomodulatory effects of MSCs are triggered via activation of TLR receptor on MSC, which is stimulated by pathogen-related molecules such as LPS or double-stranded RNA of viruses [33, 34] like HCOVID19 [13]. MSCs secrete paracrine factors, including keratinocyte growth factor (KGF), angiopoietin-1 (Ang-1), prostaglandin E2 (PGE2), interleukin 10 (IL-10), and other tropic cytokines. These paracrine factors can increase alveolar fluid clearance, regulate epithelial and endothelial permeability of the lung, facilitate endothelial repair, and reduce inflammation [35]. MSCs can also release significant quantities of extracellular vesicles (EVs) that encapsulate cytokines, growth factors, signaling lipids, mRNAs, and functional microRNAs [36]. EVs are involved in cell-to-cell communication, cellular signal transduction, and metabolism, as well as local and long-distance immune modulation [37]. In this study, due to the low immunogenicity, easy accessibility, and unique capacity of WJ-MSCs to modulate the immune system to prevent cytokine storm and inflammation caused by SARS-CoV-2, the therapeutic potential of Wharton’s jelly mesenchymal stem cells was used for patients with COVOD-19.

## Materials and methods

### Patients and study design

This pilot study was performed in Shariati Hospital of Tehran and was approved by the Ethics Committee of Tehran University of Medical Sciences (Code: IR.TUMS.VCR.REC.1399.203). This research was an open-label, single-center investigation. Patients with severe COVID-19 (according to WHO definition) were selected [38].

In this study, five patients with COVID-19 underwent cell therapy with Wharton’s jelly-derived mesenchymal stem cells. Written consent was obtained from the patients before injection. Diagnosis and management of COVID-19 patients were performed based on the WHO guidelines [5] and Iran’s diagnostic and treatment protocols. Patients were enrolled from July 21, 2020, to August 21, 2020, with the following.

#### Inclusion criteria

Inclusion criteria include the following: patients over 18 and under 65 years of age; clinical symptoms including positive COVID-19 confirmed by RT-PCR; SO2 ≤ 93% at rest or persistent hypoxia; laboratory tests CRP < 100, d-dimer< 1000, CPK twice normal levels, LDH < 245, Ferritin< 500, increasing Troponin boost and lymphopenia> 1100; respiratory failure requiring respiratory mask and involvement of over 50% of lungs with multilobar infiltrates.

#### Exclusion criteria

Exclusion criteria include the following: shift to other treatment modalities (according to the treating physician), septic shock, renal tissue insufficiency, presence of concomitant liver disorders such as increased liver enzymes or liver failure, presence of acute cardiovascular events during treatment, MI, DVT, and pulmonary embolism, enrollment in another study, and discontinuation of treatment.

#### Isolation of HWJ-MSCs

HWJ-MSC was produced by CellThecPharmed Company (Tehran, Iran) with GMP facilities approved by the food and drug administration of Iran. In brief, the umbilical cord (UC) was collected with an approximate length of 10 cm. Wharton’s jelly was isolated using a scalpel by scratching from vessels, and inner sub-amnion epithelium and the vessels were then removed. The collected WJ was placed in a separate petri dish. After the separation of Wharton’s jelly, tissue fragments were cultured in a 75-cm^2^ flask and digested by enzyme cocktails, and then a MSC cell culture medium was added to them. The flasks were incubated at 37 °C for 2–3 days without shaking, and the tissue fragments were allowed to adhere. Non-adherent cells were washed away, but adherent ones proliferated. In the next step, 80% confluency was reached after 7–10 days of growth, and the cells were transferred to another flask. Finally, cultured cells (150 × 10^6^ cells) up to passage five were used for each injection by transfer bags in GMP conditions together with heparin and human albumin serum. Confirmatory tests, including flow cytometry (CD73, CD34, CD90) and multiple lineage differentiation of MSCs (into fat, bone, and cartilage), were conducted according to the international society of cell therapy (ISCT). 150 × 10^6^ cells were considered for each patient, injected intravenously (cephalic and basilic veins) three times a week for 3 days, namely days 0, 3, and 6 for 15–20 min. Before injection, 100 mg of hydrocortisone was injected into patients to prevent complications such as allergies. Patients were then monitored on days 0, 3, 6, and 14 for routine tests, inflammatory cytokines, and flow cytometry for CD4 and CD8 markers.

All the patients were assessed for adverse events through clinical examinations, measurement of vital signs, and routine tests. Moreover, on days 0, 3, and 6, vital signs (heart rate, respiration, blood pressure, body temperature, oxygen saturation) were recorded during the injection and 1 h after it. Routine hematology parameters, myocardial enzymes, biochemistry, and inflammatory tests were performed before and after cell therapy.

SDF-1, IL-10, VEGF, TGF-β, IFN-γ, IL-6, and TNFα levels were measured in serum samples on days 0, 3, 6, and 14 (ELISA R&D, USA). COVID-19 IgM and IgG antibodies were measured the day before treatment and 14 days after it (Ideal Tashkhis Atieh, Iran).

#### Flow cytometry procedure

Peripheral blood CD4 and CD8 markers were assessed by flow cytometry. Briefly, 100 μl of whole blood was poured into three separate test tubes, each containing 10 μl of Anti-Hu CD4 PE (Exbio, Czech Republic), CD8 FITC (Exbio, Czech Republic), and Anti-Hu antibodies. CD45 PerCP (Cytognos, Spain) was mixed well and incubated at room temperature for 30 min. The red blood cells were lysed using RBC lysis buffer solution (AP-RAD, Iran) for 5 min at 300*g*. The supernatant was then discarded, and the cells were suspended with 0.3–0.5 ml PBS. The samples were immediately read using flow cytometry (Sysmex Partec Pas III, Germany).

### Statistical analysis

Some frequency tables and graphs were used to describe the individual data. The data were analyzed using the statistical package IBM SPSS version 26.0 (Statistical Package for the Social Sciences, Chicago, IL). The categorical variables are expressed as proportions and frequencies. Kolmogorov-Smirnov test was applied to test the normality distribution. To explore the independent nature of some categorical variables, a chi-square test was used. The comparison of the means between two related groups was made by paired T-test or Wilcoxon signed ranks. Generalized estimation equation (GEE) analysis was applied to test the effect of time on longitudinal data.

## Results

In this research, patients underwent cell therapy using WJ-MSC. All patients needed oxygen masks, and four of them were admitted to ICU, and one patient remained out of ICU. All patients received common treatments, including heparin and dexamethasone. Preliminary characteristics of patients such as age, sex, weight, initial baseline symptoms, duration of symptoms before hospitalization and first cell injection, ICU hospitalization period following cell injection, comorbidities, disease severity, and common drugs are listed in Table 1. All the patients were monitored for vital signs such as pulse, respiration, heart rate, blood pressure, SO_2_, and fever at the time of injection and 1 h later. In this study, no serious complication associated with WJ-MSC stem cells was observed. Only patient no. 3 had a slight post-injection headache that resolved without any drug after half an hour. The above statements indicate that WJ-MSC is safe and tolerable for the patient. There was zero mortality rate within the first 28 days.

**Table 1:** Demographic data

Patient ID	Patient 1	Patient 2	Patient 3	Patient 4	Patient 5
Gender	Female	Female	Male	Male	Male
Age	51	51	54	45	53
Weight	94	70	88	95	102
**Initial vital signs (base)**					
RR breaths/min	22	28	42	36	28
PR beats/min	71	92	89	66	92
Sys BP, mmHg	141	103	124	139	138
Dias BP, mmHg	76	71	89	90	97
Temp > 37.3 (°C, baseline)	37	37.3	36.7	37.4	36.2
SO_2_ (without oxygen mask)	87	70	79	80	89
**Other**					
COVID type	Sev	Sev	Sev	Sev	Sev
Comorbidities	Fatty liver disease	HTN	Damaged lung	No	Diabetes
Interval between symptoms onset and admission (days)	6	10	11	13	4
Interval between symptoms onset and first cell injection (days)	10	14	13	17	7
Duration of hospitalization in ICU after cell injection	7	5	0	6	0
Conventional therapy	Hep/Dexa	Hep/Dexa	Hep/Dexa	Hep/Dexa	Hep/Dexa/Ataz
RT-PCR at admission	+	+	+	+	+

*Sev* severe, *HTN* hypertension, *Hep* heparin, *Dexa* dexamethasone, *Ataz* atazanavir, *RT-PCR* reverse transcription polymerase chain reaction

**Fig. 1:**
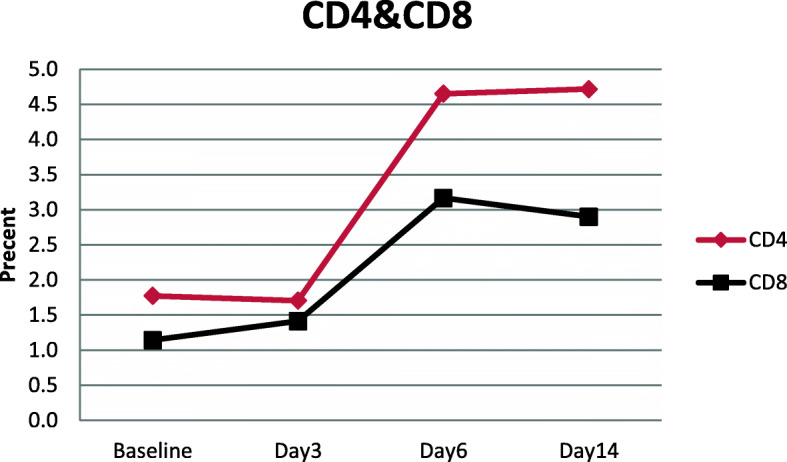
Measured markers of CD4 and CD8 on patients with COVID-19 before and after treatment

Flow cytometry analysis was performed on patient samples before and after cell therapy with WJ-MSC, and the upward trend of CD4 and CD8 markers is shown in Fig. 1, in which the improvement of lymphocyte population can be observed (more details of these markers are listed in the supplementary data file, i.e., Fig.S1a & b). In this study, the percentage of lymphocytes, absolute lymphocyte count, and CD4 and CD8 T cell ratio increased, indicating the improvement in immune system function after cell therapy with WJ-MSC (Table 2). Routine hematology tests (WBC, Hb, PLT, neutrophil and lymphocyte percentage, absolute lymphocyte count, ESR, and D-dimer), myocardial enzyme tests (CPK, LDH, and Troponin I), biochemical tests (ALT, AST, Cr, BUN, total and direct bilirubin, K, Na, and ferritin), and inflammation tests (ESR, CRP, and procalcitonin) were conducted for all patients before and after cell therapy on base and days 3, 6, and 14, which are shown in Table 2. The results indicated the improvement of test results over time. We statistically examined four of these tests that are more important for five COVID-19 patients of our study, including LDH, CRP, lymph count, and ferritin, among which ferritin showed a significant decrease (supplementary data, Fig. S2).

**Table 2:** Laboratory data

		Patient 1				Patient 2				Patient 3				Patient 4				Patient 5			
Variables	Normal Range	Base line	Day3	Day6	Day14	Base line	Day3	Day6	Day14	Base line	Day3	Day6	Day14	Base line	Day3	Day6	Day14	Base line	Day3	Day6	Day14
Routine blood tests																					
WBC count (× 10^9^/L)	3400–12,500	10940	14910	16710	14220	9600	7560	10620	8050	10400	11040	10950	11630	6100	12740	10050	9040	15106	14870	20850	24500
Hb (g/L)	M: 14–18 F: 12–16	12.1	12.7	15.1	13.7	11.5	11	11	13.5	13.1	14.2	13.5	15.1	14.7	15.1	14.4	13.8	13	13.4	14	9.3
PLT count (× 109/L)	150,000–450,000	146	195	227	218	233	321	428	295	287	243	192	205	307	328	183	189	423	501	534	269
Neutrophil (%)	45–75	NA	88	88	70	90	80	83	62	90	82	70	71	86	91	71	81	97	87	98	94
Lymphocyte (%)	20–40	NA	2	10	19	4	9	11	26	5	10	20	20	8	2	21	14	3	5	2	2
LYM count (× 10^9^/L)	1.0–4.0	NA	298.2	1671	2701.8	384	680.4	1168.2	2093	520	1104	2190	2326	640	254.8	2110.5	1265.6	453.18	743.5	417	490
ESR (mm/h)	M: 0 to 20 F: 0 to 25	96	73	23	26	104	NA	NA	51	74	9	25	10	37	5	64	17	80	50	NA	61
D-Dimer (ng/ml)	Up to 442	441	481	436	416	2071	1573	869	589	779	1234	11302	13321	15000>	12540	13214	10222	11321	11206	10278	9278
Myocardial enzymes																					
CPK (U/L)	5 to 25	21.5	38	10	16.8	16	3.23	7.7	17.6	61	249	38	22	104	125	34	34	128	232	119	65
Troponin I (ng/ml)	≤ 0.4	0.1	1.4	1.4	0.1	3.4	1.06	0.88	0.72	4.5	0.4	0.6	0.84	3.7	1.5	0.8	0.9	1.3	4.4	0.67	17.6
LDH (U/L)	240–480	723	939	462	374	860	483	427	545	542	465	392	398	1458	1117	615	417	987	1182	1628	906
Biochemical indicators																					
Total Bili (mg/dl)	0.1–1.2	0.26	0.7	1	0.6	1.1	0.54	0.43	0.9	0.7	0.6	0.7	1.3	1.6	1.6	1.6	0.9	4.55	4.3	2.6	1.3
Direct Bili (mg/dl)	Up to 0.3	0.1	0.3	0.3	0.1	0.3	0.2	0.02	0.1	0.1	0.1	0.2	0.2	1.2	0.6	0.3	0.2	0.41	0.8	0.6	0.3
ALT (U/L)	M: up to 41 F: up to 31	55	29	40	43	98	41	42	39	16	43	30	11	136	106	55	75	66	20	19	20
AST (U/L)	M: up to 38 F: up to 31	23.5	20	25	20	68	47.5	25	18	19	40	15	35	145	50	29	27	101	30	25	25
BUN (mmol/L)	7–20	6	23	24	16	23	54	43	21	25.2	16	21	21	25	14	11	11	18	20	20	25
Cr (μmol/L)	M: 0.7–1.4 F: 0.6–1.3	0.8	0.8	0.7	0.8	0.5	0.9	1	1.1	0.9	0.8	1.1	0.9	0.8	0.7	0.8	0.7	1.03	0.9	0.9	1.1
K (mmol/L)	3.6–5.2	4.1	4.8	4.8	NA	4.1	3.7	4.4	4.1	4.7	4.4	4.5	4.9	4.8	4.1	4.5	4.5	4.7	4.2	4.6	4.9
Na (mEq/L)	135–145	139	137	138	NA	144	143	139	145	144	142	141	145	144	137	142	145	141	136	139	138
Inflammatory markers																					
CRP (mg/L)	Up to 10	60.5	6	3	0.48	107	101	20	3.84	87	53.5	24.5	4.44	45	82	35	1.47	60	29.5	72.5	49
Procalcitonin (ng/ml)	< 0.10	0.17	0.01	0.03	0.08	0.02	0.12	0.07	0.03	0.11	0.09	0.07	0.05	0.1	0.11	0.13	0.02	0.04	0.33	0.1	0.18
Ferritin (ng/ml)	M: 24 to 336 F: 11 to 307	1979	959	231	169	798	986	686	659	896	707	600	500	1087	2666	742	878	2314	1181	656	828

In this study, the trend of oxygen saturation percentage among patients over MSC injection days (admission day, days 0, 3, and 6; supplementary data, Fig. S3-a), at the time of injection, and 15, 30, 45, and 60 min after it is shown in Fig. 3-b (supplementary data).

**Fig. 2:**
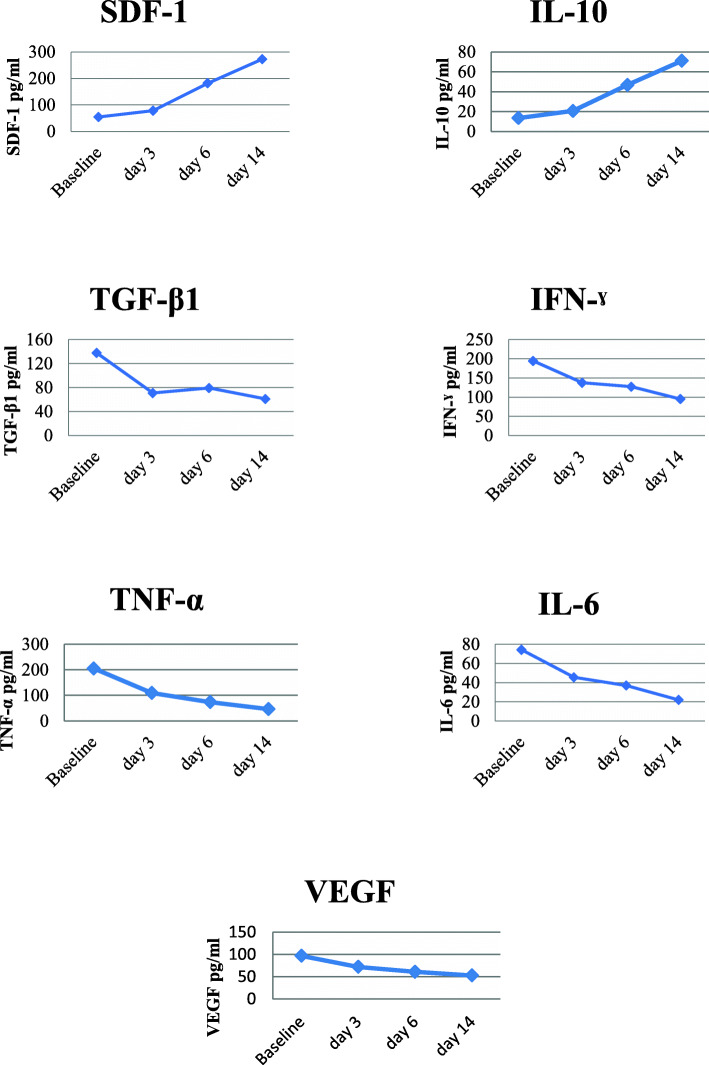
Inflammatory cytokines. SDF-1 : stromal cell-derived factor 1, IL-10 : Interleukin 10, TGF-β1 : Transforming growth factor beta 1, IFN ˠ : Interferon gamma, TNF-α : Tumor necrosis factor alpha,IL-6 : Interleukin 6, VEGF : Vascular endothelial growth factor

In this research, we examined inflammatory cytokines, angiogenesis, and implantation in the days before and after cell therapy, as shown in Fig. 2. The results show that over 14 days before and after cell therapy, SDF-1 and IL-10 levels increased, but VEGF, TGF-β, IFN-γ, IL-6, and TNFα decreased.

COVID-19 antibody test in the days before treatment and 14 days after it are shown in Fig. S4 (Supplementary data, Fig. S4).

### CT scan

**Fig. 3:**
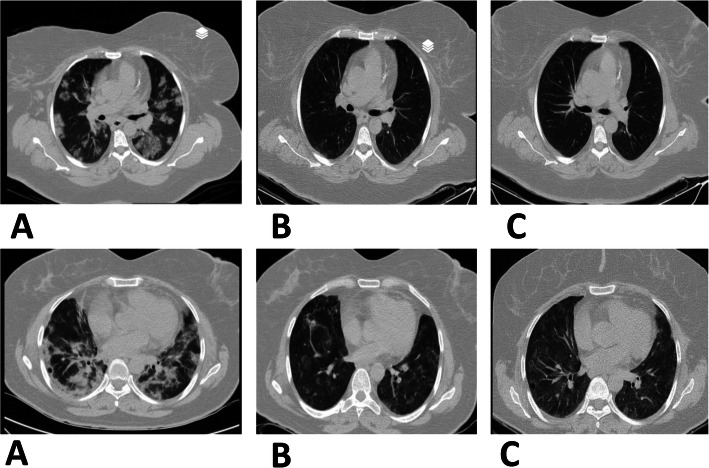
Lung CT scan. **a** (A–C) There are multifocal patchy alveolar opacities in both lungs that in follow-up exam 14 days and 30 days later disappeared following stem cell therapy. P1: Patient 1. **b** (A–C) There are multifocal patchy alveolar opacities in both lungs that in follow-up exam 14 days and 30 days later disappeared following stem cell therapy. P2: Patient 2

Table 3 lists CT scan results of parenchymal abnormalities, type of GGO opacities, zonal involvement, total score, and cardiomegaly. None of the patients had pleural effusion. One patient showed mild (patient no. 2) pericardial effusion before cell injection and14 days after it but was free of pericardial effusion on day 28. Mild P4 emphysema was observed on days 14 and 28. None of the patients showed pulmonary fibrosis. Patient no. 5 showed mild bronchiectasis the day before cell therapy and at day 14. All patients (except for patient no. 3) showed mild to moderate cardiomegaly. In patient no. 1, the mosaicism pattern was completely resorbed on day 28, patient P2 showed severe mosaicism on day 28, patient no. 3 had moderate mosaicism on day 14 and mild mosaicism on day 28, and finally, patient no. 4 showed severe mosaicism on days 14 and 28. Patient no. 5 had no mosaicism. Chest computerized tomography (CT) images of the two COVID-19 patients are demonstrated in Fig. 3a, b.

**Table 3:** CT evaluation

Parameters	Patient- 1 (WJ-MSC treatment)			Patient- 2 (WJ-MSC treatment)			Patient- 3 (WJ-MSC treatment)			Patient- 4 (WJ-MSC treatment)			Patient- 5 (WJ-MSC treatment)		
	Base	Day 14	Day 30	Base	Day 14	Day 30	Base	Day 14	Day 30	Base	Day 14	Day 30	Base	Day 14	Day 30
• **CT parenchymal abnormalities:**															
a- GG0	b	a ,c	a	a,b,d,e	a,c,d,e	a, c	a,c	a,c,d	a	b	a,b,e	a,e	a,b,d,g	a,b,d	NA
b- Consolidation															
c- Reticular pattern															
d- Mixed pattern															
e- Linear opacities															
f- Multifocal GGO of rounded morphology															
g- Reverse halo sign or other findings of organizing pneumonia															
•** Type of GGO opacities:**															
a- Pure GGO															
b- Crazy paving: GGO + intralobular lines	a	a	a	d	c,d	d	a	a,d	d	a	a,d	a,d	b	a	NA
c- Irregular lines and interfaces with architectural distortion + GGO															
d- Streaky pattern															
• **Zonal involvement:**															
a- Right upper: (0–5% = 1, 5–25% = 2, 26–50% = 3, 51–75% = 4, 76–100% = 5)	a (3)	a (2)	a (0)	a (3)	a (4)	a (3)	a (2)	a (3)	a (2)	a (4)	a (5)	a (1)	a (2)	a (5)	NA
b- Right middle: (0–5% = 1, 5–25% = 2, 26–50% = 3, 51–75% = 4, 76–100% = 5)	b (3)	b (2)	b (0)	b (4)	b (2)	b (2)	b (2)	b (3)	b (1)	b (4)	b (5)	b (2)	b (3)	b (5)	
c- Right lower: (0–5% = 1, 5–25% = 2, 26–50% = 3, 51–75% = 4, 76–100% = 5)	c (3)	c (1)	c (0)	c (4)	c (2)	c (2)	c (2)	c (3)	c (2)	c (4)	c (3)	c (2)	c (3)	c (5)	
d- Left upper: (0–5% = 1, 5–25% = 2, 26–50% = 3, 51–75% = 4, 76–100% = 5)	d (3)	d (2)	d (1)	d (3)	d (4)	d (3)	d (2)	d (3)	d (2)	d (4)	d (5)	d (1)	d (3)	d (5)	
e- Left middle: (0–5% = 1, 5–25% = 2, 26–50% = 3, 51–75% = 4, 76–100% = 5)	e (3)	e (1)	e (0)	e (4)	e (2)	e (2)	e (2)	e (3)	e (1)	e (4)	e (5)	e (2)	e (4)	e (5)	
f- Left lower: (0–5% = 1, 5–25% = 2, 26–50% = 3, 51–75% = 4, 76–100% = 5)	f (3)	f (2)	f (1)	f (4)	f (3)	f (2)	f (3)	f (3)	f (2)	f (4)	f (3)	f (2)	f (3)	f (5)	
• **Total score of zonal involvement in both lungs:**	18	10	2	22	17	14	13	18	10	24	26	10	18	30	NA
• **Cardiomegaly:** (Yes/No) Severity (mild, moderate, severe)	Yes (mild)	Yes (mild)	Yes (mild)	Yes (mild)	Yes (mild)	Yes (mild)	No	No	No	Yes (mild)	Yes (mild)	Yes (mild)	Yes (mild)	Yes (mild)	NA
a. Highly suggested															
b. Indeterminate	a	b	d	a	b	c	a	a	b	a	b	b	a	a	NA
c. Inconsistent															
d. None

## Discussion

Most patients show a tolerant response to COVID-19 infection. When the virus enters the lungs, immune system cells are recalled to the infected area to defend the body against the virus and elicit an immune response [39]. In some cases, the increase in cytokines secreted by this response leads to cytokine storm, followed by inflammation, tissue damage, secondary infection, and ARDS, leading to death [40, 41]. Therefore, inhibition of cytokine storms in patients with COVID-19 can be crucial in treating these patients [24].

Studies have revealed that mesenchymal stem cells are involved in improving lymphocyte populations mainly through dendritic cells and shifting immune system cells’ response from Th1 to Th2 [42]. TCD8 cells are of high importance in killing virus-infected cells [43]. TCD4 cells play an essential role in the immune response by helping TCD8 and B cells to produce antibodies [44]. Various investigations have indicated that in patients with severe COVID-19, both TCD4 and TCD8 are reduced [45]. In our study, both TCD4 and TCD8 cells were recovered after cell therapy. A clinical trial was conducted in China on seven patients with COVID-19 pneumonia injected with mesenchymal stem cells. Patients who showed no improvement in symptoms compared to conventional treatment were recruited [13]. MSCs inhibit the overactivation immune system and promote endogenous repair by improving the microenvironment. In this study, it was stated that after IV injection of MSCs, these cells accumulated in the lungs and could improve the pulmonary microenvironment, prevent pulmonary fibrosis, and improve lung function [13]. According to Cao team reports, serum levels of IL-2, IL-7, GCSF, IP10, MCP1, MIP1A, and TNFα were considerably higher in ICU patients compared to ordinary people [12]. This research showed that IV injection of MSCs improves inflammation in patients with severe COVID-19. Besides, increasing IL-10 and VEGF promotes lung repair [13]. In a study by Lanzoni et al., the safety and, to some extent, the efficacy of hUC-MSC cells in ARDS patients induced by COVID-19 was evaluated. Patients received 100 ± 20 × 10^6^ cells in two doses intravenously. They took drugs such as remdesivir, corticosteroids, hydroxychloroquine, and tocilizumab together with the cells. In this study, they stated that hUC-MSC cells are safe in these patients and significantly reduce side effects and death and improve recovery time compared to the control group [46]. In another research by Hashemian et al., both PL-MSC and UC-MSC cells were used in ARDS patients. Patients received 200 × 10^6^ cells at three doses through intravenous infusion with IVIG, ribavirin, and favipiravir. In these patients, factors such as IL-6, IL-8, TNFα, INF-γ, IL-4, IL-10, and CRP were also evaluated. This research stated that placental and umbilical cord stem cell injections are safe and can quickly improve respiratory symptoms reducing inflammatory factors in several patients [47].

SDF-1 factor is a cytokine that plays a crucial role in organ formation and repair after injury [48]. In a number of MSC implantation studies, it has been shown that increasing SDF-1 levels after tissue injury plays an essential role in the accumulation of mesenchymal stem cells at the site of injury [49–51]. Increased SDF-1 also increases CXCR4 expression on mesenchymal stem cells and plays a vital role in transplantation of these cells into damaged tissue [52]. Another study indicated that using SDF-1 along with WJ-MSC cells increased the migration of these cells in vitro [53]. VEGF is a significant factor in acute lung injury and ARDS, and the increase of that can be seen in acute inflammation and hypoxia [54, 55]. In addition to angiogenesis, VEGF increases vascular leakage and permeability [56, 57]. Studies have indicated that VEGF concentrations increase in patients with COVID19 admitted to the ICU and those outside it [3]. In our research, VEGF was gradually reduced after WJ-MSC cell therapy. In one of our patients (no. 1) in the days before cell therapy, there was a bloody sputum complaint, which may be due to increased level of VEGF and subsequently increased permeability of small pulmonary arteries. The rise in VEGF level was more prominent in this patient than in other patients, which resolved after receiving the first dose of cells within 24 h. Mesenchymal stem cell injections reduce TGF-β, IFN-γ, and TNFα levels in the lung. In our study, similar to others [58, 59], inflammatory factors including IL-6, TNFα, and related factors associated with pulmonary fibrosis were reduced, including TGF-β and IFN-γ. In this research, it seems that the time of cell injection in these patients is of high importance, and it can be stated that the optimal time for cell injection is the second week of the disease, namely the second phase of disease or hyper inflammation [60] beginning on days 7 to 15.

## Conclusions

This study showed that the injection of Wharton’s jelly-derived stem cells was safe and well-tolerated by the patient. From our perspective, the timing of stem cell injections in patients with severe COVID19 is critical. It seems that it is better to inject these cells in the inflammatory phase, and for this purpose, we should check inflammatory tests for the patient before cell injection. Considering the behavior of mesenchymal stem cells, it seems that paying attention to the precise protocols of isolation, culture, proliferation, an appropriate number, manner, and time of proper injection into humans can be the beginning of a new treatment strategy in COVID19. Further studies should be conducted to prove the effective outcomes using control and treatment groups to indicate these significant differences. It is also necessary to increase the sample size and use randomization methods in further studies to indicate these cells’ positive function and improve the disease.

## Supplementary Information

**Additional file 1: Figure S1.** flowcytometry. a. Patient 2, Lymphocyte regeneration, A. control, B. base (before WJ-MSC injection), C.day3, D.day6 and E. day 14 after WJ-MSC injection. b. Patient 4, Lymphocyte regeneration, A. control, B. base (before WJ-MSC injection), C.day3, D.day6 and E. day 14 after WJ-MSC injection. **Figure S2.** laboratory data. GEE analysis was also applied to show the effect of time on change of some main variables such as CRP, Lymph count, Ferritin and LDH. The results showed a significant change during time just for ferritin with p-value, 0.008. **Figure S3.** O2 Saturation. Generalized Estimating Equation (GEE) Analysis. GEE modeling was used to show the effect of day (0, 3 and 6) and time of injection (start, min15, min30, min45 and min60) on O2sat change. Baseline values were entered to the GEE model as a covariate variable .The overall mean O2sat during three days had a same trend but in return, injection time has a significant effect on O2sat as mean O2sat at start is different to other injection time (p-value=0.001). Mean O2sat at injection time is: 91.3, 92.3, 92.3, 92.5 and 93.1. a. Mean of O2sat during time. b. Change of O2sat by patients. **Figure S4.** SARSCOV2 Abs. Paired Comparison Analysis. Mean SARSCOV2.IgM and SARSCOV2.IgG at baseline and end of study, were compared using Wilcoxon Signed-Ranks. The line charts related to both antibodies are shown in Fig.S4.

## Data Availability

All of the data generated and analyzed during this study are included in our manuscript.
