# Supplementary material for: Cell therapy in patients with COVID-19 using Wharton’s jelly mesenchymal stem cells: a phase 1 clinical trial
Source: Stem Cell Res Ther. 2021 Jul 16;12:410. doi: 10.1186/s13287-021-02483-7 (PMC8283394; doi:10.1186/s13287-021-02483-7)
Supplement: Supplementary file 1 — Additional file 1: Figure S1. flowcytometry. a. Patient 2, Lymphocyte regeneration, A. control, B. base (before WJ-MSC injection), C.day3, D.day6 and E. day 14 after WJ-MSC injection. b. Patient 4, Lymphocyte regeneration, A. control, B. base (before WJ-MSC injection), C.day3, D.day6 and E. day 14 after WJ-MSC injection. Figure S2. laboratory data. GEE analysis was also applied to show the effect of time on change of some main variables such as CRP, Lymph count, Ferritin and LDH. The results showed a significant change during time just for ferritin with p-value, 0.008. Figure S3. O2 Saturation. Generalized Estimating Equation (GEE) Analysis. GEE modeling was used to show the effect of day (0, 3 and 6) and time of injection (start, min15, min30, min45 and min60) on O2sat change. Baseline values were entered to the GEE model as a covariate variable .The overall mean O2sat during three days had a same trend but in return, injection time has a significant effect on O2sat as mean O2sat at start is different to other injection time (p-value=0.001). Mean O2sat at injection time is: 91.3, 92.3, 92.3, 92.5 and 93.1. a. Mean of O2sat during time. b. Change of O2sat by patients. Figure S4. SARSCOV2 Abs. Paired Comparison Analysis. Mean SARSCOV2.IgM and SARSCOV2.IgG at baseline and end of study, were compared using Wilcoxon Signed-Ranks. The line charts related to both antibodies are shown in Fig.S4. [file 13287_2021_2483_MOESM1_ESM.docx]

**Supplementary data file for**

**Cell Therapy in Patients with COVID-19 Using Wharton’s Jelly Mesenchymal Stem Cells: A Phase 1 Clinical Trial**

**Mahshid Saleh , Amir abbas vaezi , Rasoul Aliannejad, Amir Ali Sohrabpour, Seyedeh Zahra Fotook Kiaei, Mahdi Shadnoush, Vahid Siavashi, Leila Aghaghazvini, Batoul Khoundabi, Shahriyar Abdoli, Bahram Chahardouli, Imman seyhoun*, Neda alijani*, Javad Verdi***

**Corresponding authors**

**Dr. Javad Verdi. PhD**

Javad Verdi, Department of Applied Cell Sciences, School of Advanced Technologies in Medicine, Tehran University of Medical Sciences, Tehran, Iran.

Email: javadverdi2019@gmail.com

**Dr. Neda Alijani. MD**

Department of lnfectious Diseases,Shariati Hospital,Tehran University of Medical Sciences,Tehran,Iran.

Email: [n-alijani@sina.tums.ac.ir](mailto:n-alijani@sina.tums.ac.ir)

<https://orcid.org/0000-0002-7506-811X>

**Dr. Imman seyhoun . PhD**

Department of Applied Cell Sciences, School of Advanced Technologies in Medicine, Tehran University of Medical Sciences, Tehran, Iran, School of Advanced Technologies in Medicine, Tehran University of Medical Sciences, Tehran, Iran.

Email: [i.seihoon@gmail.com](mailto:i.seihoon@gmail.com)

NA, IS, JV contributed equally to this work.

**Supplementary data file include Fig S1 to S4**

**Fig.S1 flowcytometry**


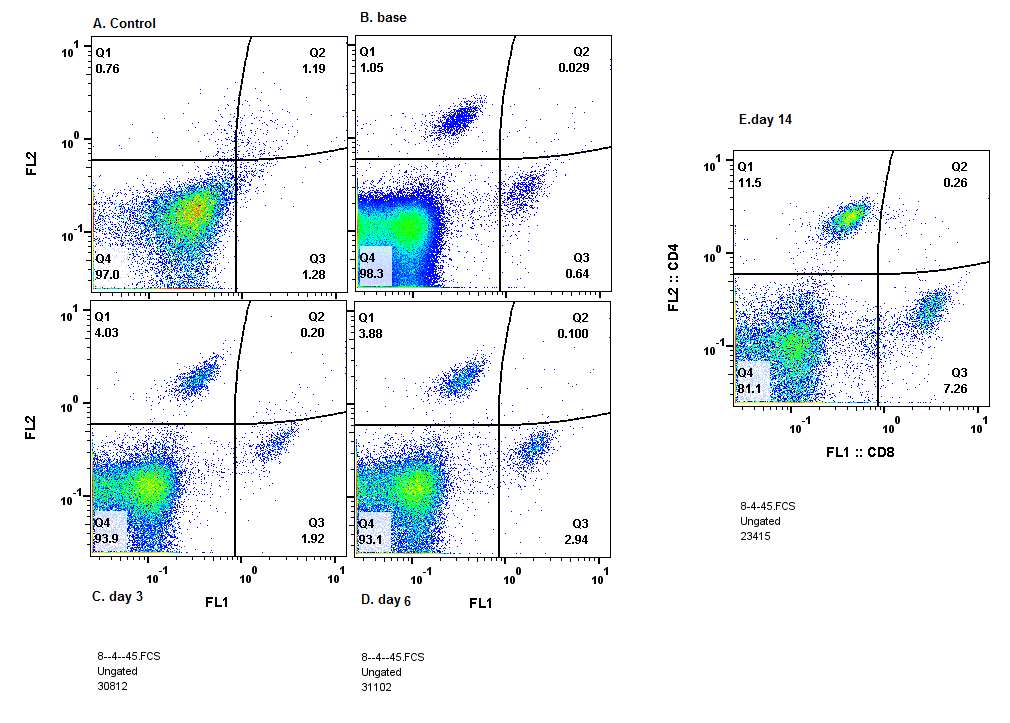


Fig S1 a. Patient 2, Lymphocyte regeneration, A.control, B. base (before WJ-MSC injection), C.day3, D.day6 and E.day 14 after WJ-MSC injection.


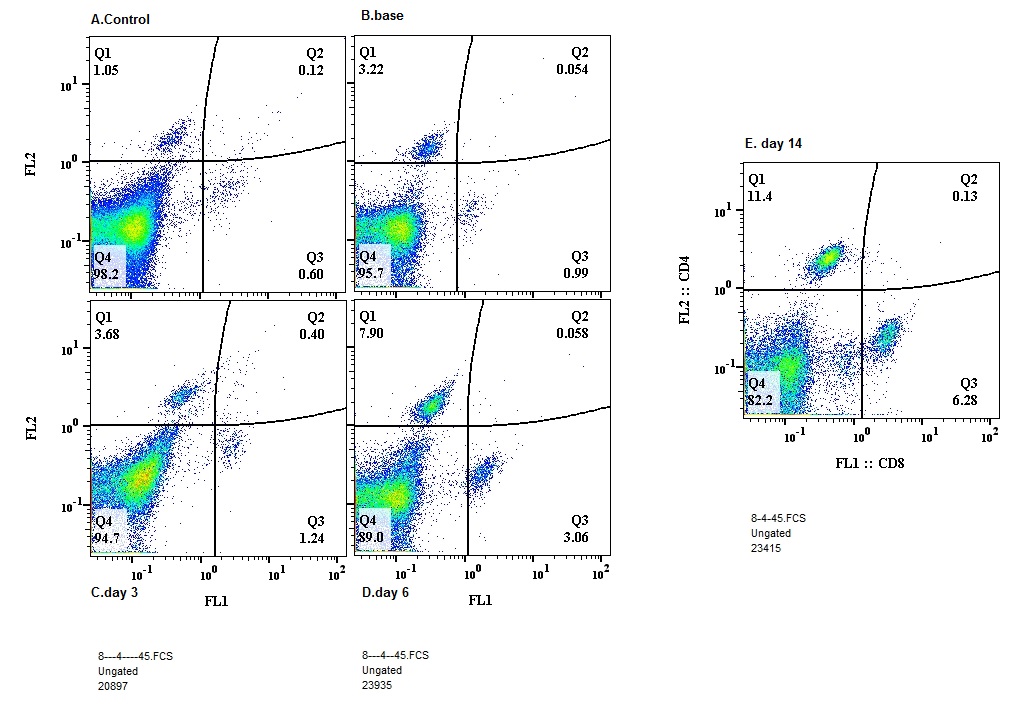


Fig S1 b. Patient 4, Lymphocyte regeneration, A.control, B. base (before WJ-MSC injection), C.day3, D.day6 and E.day 14 after WJ-MSC injection.

**Fig. S2 laboratory data**

GEE analysis was also applied to show the effect of time on change of some main variables such as CRP, Lymph count, Ferritin and LDH. The results showed a significant change during time just for ferritin with p-value, 0.008.

| Fig. S2 laboratory data | |
| --- | --- |
|  |  |
|  |  |

**Fig. S3 O2 Saturation**

**Generalized Estimating Equation (GEE) Analysis**

GEE modeling was used to show the effect of day (0, 3 and 6) and time of injection (start, min15, min30, min45 and min60) on O2sat change. Baseline values were entered to the GEE model as a covariate variable .The overall mean O2sat during three days had a same trend but in return, injection time has a significant effect on O2sat as mean O2sat at start is different to other injection time (p-value=0.001). Mean O2sat at injection time is: 91.3, 92.3, 92.3, 92.5 and 93.1.

Fig.S3-a.Mean of O2sat during time

|  |  |  |
| --- | --- | --- |

Fig.S3-b.Change of O2sat by patients

**Fig. S4 SARSCOV2 Abs**

**Paired Comparison Analysis**

Mean SARSCOV2.IgM and SARSCOV2.IgG at baseline and end of study, were compared using Wilcoxon Signed-Ranks. The line charts related to both antibodies are shown in Fig. S4.

Fig. S4. COVID-19 IgM & IgG Ab
